# Trials, registries and guidelines for non-ST-elevation acute coronary syndromes

**DOI:** 10.1007/s12471-013-0495-7

**Published:** 2013-11-21

**Authors:** F. W. A. Verheugt

**Affiliations:** Department of Cardiology, Onze Lieve Vrouwe Gasthuis (OLVG), PO Box 95500, 1090 HM Amsterdam, the Netherlands

The most common admission indication in cardiology practice is acute coronary syndrome with or without ST-segment elevation. In patients presenting with ST-segment elevation at admission, ECG reperfusion therapy is instituted as fast as possible [1–3], which can be accomplished by primary PCI, by fibrinolysis, or both. This results in a significant reduction in infarct size and improvement of short- and long-term prognosis [2, 3]. In patients with coronary syndromes presenting without ST-segment elevation on the admission ECG, anti-ischaemic and antithrombotic therapies are also of utmost importance. However, in the last decade a strong switch has been seen in the invasive approach to this condition. Angiography can improve risk stratification and, if indicated, revascularisation can be planned. Several randomised trials have evaluated a routine invasive strategy in comparison to a more selective invasive approach in these patients. The outcome results were mixed [4–6]. Although these meta-analyses included trials from the pre-clopidogrel era, a routine invasive strategy showed a reduction in myocardial infarction and repeat intervention. The results with regard to early and long-term survival were also variable. There seemed to be an early hazard for early mortality compensated by a later reduction [4].

The substrate of a non-ST-elevation myocardial infarction does not usually consist of an acutely occluded epicardial coronary artery as in ST-segment-elevation myocardial infarction. In non-ST-elevation acute coronary syndromes (NSTE-ACS) there can be severe but non-occlusive coronary artery disease, or no disease at all. In the large TACTICS-TIMI-18 study nearly half of the patients had left main or triple vessel disease and only 13 % had normal coronary arteries [7]. In both instances reperfusion therapy is not indicated and only leads to harm by bleeding and excess myocardial infarction [8, 9].

The cornerstone of the treatment of patients undergoing coronary stenting for acute coronary syndromes is dual antiplatelet therapy with aspirin and the platelet P_2_Y_12_ receptor antagonist clopidogrel. Consequently, many patients in cardiology practice in 2013 are on dual antiplatelet therapy, mainly aspirin and clopidogrel. The only important side effect of dual antiplatelet therapy is increased bleeding in comparison with aspirin alone. This has been established in the large trials with clopidogrel in ACS [10, 11] and thereafter [12], also in atrial fibrillation [13]. Especially in the latter dual antiplatelet therapy has shown to be as hazardous as oral anticoagulation [14]. The novel platelet P_2_Y_12_ receptor antagonists prasugrel and ticagrelor are more effective than clopidogrel in patients, but show more major bleeding including intracranial haemorrhage [15, 16]. Prasugrel is specifically indicated and registered for use after PCI for ACS.

In today’s issue of the Netherlands Heart Journal, the design of a study on adherence to evidence-based medicine in a large group of patients discharged after ACS is described [17]. The strength of the paper is that the study is (a) carried out in a large single region, (b) prospective in nature and (c) part of a thorough registry. In this registry the new oral antiplatelet drug prasugrel will be used. The interim results of this study are now available online and will be published in a forthcoming edition of the journal [18].

Of course, the weakness of the registry is the potential confounding bias, which is inherent to every registry, even the prospective ones. A randomised comparison with e.g. ticagrelor or even clopidogrel would, therefore, be preferable, but randomised controlled trials have the severe shortcoming of selection bias (Table 1). For a long time now, meta-analyses have been used to evaluate treatment effects of certain medical strategies in a broader perspective. However, meta-analyses not only suffer from publication bias and, therefore, may overestimate a treatment effect, they often also show considerable heterogeneity. Unfortunately, many guidelines appreciate meta-analyses as level of evidence similar to that of individual randomised controlled trials. Given the above, this must be discouraged. At best, meta-analyses are hypothesis generating, and should not be used to underscore the weight of a cluster of individual randomised trials. Even in the absence of properly randomised studies with enough power, meta-analyses should be omitted from guidelines when it comes to weighing level of evidence. Both randomised studies and registries for medical procedures are of the utmost importance. Although clinical trialists despise observational studies because of the confounding factors, they should admit that they represent the real world provided all incident NSTE-ACS cases are entered into the registries For that purpose, only prospective registries are acceptable for therapy evaluation. The current design paper meets this criterion.

**Table 1:** Shortcomings of randomised trials, meta-analyses, registries and guidelines

Method	Shortcomings
Randomised trial	Selection bias
	Poor generalisibility
Meta-analysis	Publication bias
	Overestimation of treatment effect
	Often considerable heterogeneity
Registry	Confounding bias
Guideline	Meta-analyses used as high level of evidence

In conclusion, the design of a registry presented in this issue is a solid basis for the collection of evidence-based discharge strategies after ACS. However, a new antiplatelet drug will be used in the registry for the patients treated with PCI for their index ACS. This may be a confounded registry, in that prasugrel can be used exclusively in patients without contraindications to the agent (age, prior stroke and low body weight). In that case clopidogrel or ticagrelor may be preferred. And hopefully pre-treatment with prasugrel will be avoided, since it is not helpful and causes increased bleeding [19]. But given the excellent past clinical performance of the study group it will implement the most recent study data into their practice.
